# Biomass allocation and seasonal non-structural carbohydrate dynamics do not explain the success of tall forbs in short alpine grassland

**DOI:** 10.1007/s00442-021-04950-7

**Published:** 2021-05-28

**Authors:** Erika Hiltbrunner, Jonas Arnaiz, Christian Körner

**Affiliations:** Department of Environmental Sciences, Institute of Botany, University of Basel, Schönbeinstrasse 6, 4056 Basel, Switzerland

**Keywords:** Functional growth analysis, High-elevation, Microclimate, Stature, ^13^C labelling

## Abstract

The majority of alpine plants are of small stature. Through their small size alpine plants are decoupled from the free atmospheric circulation and accumulate solar heat. However, a few alpine species do not follow that “rule” and protrude with their aboveground structures from the microclimatic shelter of the main canopy boundary layer. We aim at explaining the phenomenon of being tall by exploring the biomass production and carbon relations of four pairs of small and tall phylogenetically related taxa in alpine grassland. We compared species and stature-specific biomass allocation, shifts in non-structural carbohydrate (NSC) concentrations in different tissues throughout the season, and we used ^13^C labels to track carbon transfer from leaves to belowground structures. Small and tall herbs did not differ in their above- to belowground biomass allocation. The NSC composition (starch, fructan, simple sugars) and allocation did not show a stature-specific pattern, except for higher concentrations of simple sugars in tall species during their extended shoot growth. In relative terms, tall species had higher NSC pools in rhizomes, whereas small species had higher NSC pools in roots. Our findings do not place tall alpine forbs in an exceptional category in terms of biomass allocation and carbohydrate storage. The tall *versus* small stature of the examined herbs does not seem to be associated with specific adjustments in carbon relations. ^13^C pulse labelling revealed early C autonomy in young, unfolding leaves of the tall species, which are thus independent of the carbon reserves in the massive belowground organs.

**Supplementary Information:**

The online version contains supplementary material available at 10.1007/s00442-021-04950-7.

## Introduction

In the temperate zone, life conditions of alpine plants are characterised by long, cold winters with a protective snow cover, and a relatively short growing season (2.5–3.5 months). The actual climate alpine plants commonly experience during the growing season is not well represented by air temperature measured at a weather station (Körner and Larcher 1988; Scherrer et al. 2011; Körner and Hiltbrunner 2018). Owing to their small stature and dense canopies, most alpine plants operate within an aerodynamic boundary layer that decouples them thermally from free atmospheric circulation. Consequently, foliage temperatures in alpine plants are not much different from those in low elevation grassland during bright daylight hours, which is reflected in their photosynthetic temperature response (Körner and Diemer 1987). Across three alpine slopes and over an entire growing season, these microclimate benefits resemble a maximum downslope shift in elevation by 1900 m (corresponding to a 10.5 K difference in surface temperature; Scherrer and Körner 2011). Hence, most alpine plants are genotypically dwarfs and profit from the sun-driven canopy warming. Also, flower heads of many alpine species accumulate heat (Dietrich and Körner 2014), most likely with positive effects on plant reproduction including pollination and seed ripening. Yet, a few tall taxa in all alpine ecosystems (e.g., in the genera *Gentiana, Rumex, Senecio, Veratrum*) appear to disregard these benefits of small stature. Are these taxa falsifying that 'rule'? What permits tall herbs to thrive outside the protective boundary layer? Some of these tall herbs are confined to sheltered gullies or nutrient-rich, ruderal locations (e.g., *Rumex alpinus*), but others are not. Here, we explore this question with a focus on growth and carbon relations.

To build such tall structures in a very short period of time might require special biomass allocation regimes and non-structural carbon pools that can rapidly be activated and invested in growth. It is well established that biomass allocation into storage organs has a strong influence on plant growth, potentially outweighing the role of per-unit-leaf area C assimilation (functional growth analysis; Briggs et al. 1920; Körner 1991; van der Werf et al. 1993; Poorter et al. 2000).

Biomass allocation is under strong genetic control, yielding the typical species and life form specific appearance of plants (Körner 2014). For alpine and arctic herbaceous plants, such allocation patterns converge to a surprisingly similar community means but with a substantial species-specific variation (Körner and Renhardt 1987; Patty et al. 2010). Alpine herbaceous plants invest more in specific storage organs and roots, and less in stems, with the mean leaf mass fraction largely conserved compared to low elevation herbs. However, these surveys did not include tall herbs. They may adopt a contrasting investment strategy.

To our best knowledge, the majority of alpine tall herbs belong to the rhizomatous group of plants. Massive rhizomes may simply act as a space holder, contributing to the persistence of a species (Klimešová et al. 2018a, b). Whether rhizomes play a greater role in their carbon economy than in rhizomatous small herbs remains to be shown. Rhizomes in tall herbs may be more conspicuous simply for their size, but the biomass fraction may not differ from that in their small relatives. Although relatively large storage organs (up to 50% of total dry mass, Patty et al. 2010) are abundant in alpine herbs, some of the most extreme, high elevation habitats are inhabited by plant species that completely lack specific storage organs but store in the bulk of their fine roots, especially, when the rooting substrate is not stable as in recently released glacier forefields (e.g., species of *Cerastium*, *Saxifraga*; Körner and Renhardt 1987).

In perennial, rhizomatous species, the rhizomes increase in size every year, resulting in differently aged rhizome parts, and some species exhibit characteristic annual increments such as in *Rumex alpinus* (Klimeš 1992). Such special storage organs are assumed to assist in fast spring flushing through immediate remobilisation of stored carbohydrates. In addition to structural biomass allocation, the allocation and amount of non-structural carbon compounds may also contribute to tall herb success in alpine grassland. For perennial, alpine plants, storage and recycling of carbohydrates (but also other compounds such as nutrients and lipids) may be crucial to rapidly develop and survive in a short season, and high tissue concentrations of NSC have been reported, particularly for perennial tissues. It is unresolved whether these NSC deposits are simply stores of extra-photoassimilates or represent specific reserves (Chapin et al. 1990).

The most common NSC compounds are free, simple sugars of low molecular weight (glucose, fructose, sucrose), oligosaccharides, polysaccharides of variable length and degree of polymerisation and starch (Chapin et al. 1990; Hoch et al. 2003). Fructans have been shown to rise in abundance in cool environments, thus, alpine species often reveal high fructan concentrations in different tissues (Chatterton et al. 1989). Stored NSC compounds may change considerably in the different plant compartments during the growing season. In belowground organs of alpine plants, these stores may be reduced prior to, or during bud break and early-season shoot extension (Mooney and Billings 1960; Fonda and Bliss 1966; review in Körner 2021; Orthen and Wehrmeyer 2004). This early season NSC depression may become more pronounced in tall alpine herbs, and leaf autonomy may be reached later than in small herbs. Here, pulse labelling by high concentrations of enriched ^13^CO_2_ represents a simple method for tracing the export of newly assimilated CO_2_ from leaves to other organs, hence, to study leaf C autotrophy (Studer et al. 2014).

On the other hand, the NSC concentrations in large rhizomes can be relatively stable over time, even in differently aged rhizome sections (Klimeš et al. 1999), become lower with increasing rhizome age (Shaver and Billings 1976) or vice versa (Klimešová and Klimeš 1996). NSC stores may not be diminished at peak demand for growth, and are not necessarily ever used, since high concentrations have been found in very old or dead plant fractions (Menke and Trlica 1981; Körner 2021). Old C stores of the alpine herb *Oxytropis sericea* commonly not used during the growing season, were remobilised when plants were experimentally shaded, indicating that these stores became 'reserves' in an emergency (Wyka 1999). Hence, it depends on life conditions or on the degree of disturbance whether stores are reserves (Chapin et al. 1990).

In the present study, we test three specific hypotheses explaining why and how tall alpine herbs are capable to thrive outside the boundary layer of the commonly small alpine vegetation.

1. Though tall herbs reach a higher plant height and obviously produce more aboveground biomass at peak season than small herbs, they may have a preferential dry mass allocation to belowground organs (sensu functional growth analysis) due to their dependence in rhizomes.
2. The tall stature strongly relies on high NSC provision during growth, a pronounced remobilisation from rhizomes or storage roots and transfer to the rapidly expanding above-ground tissues and refilling of the reserves at the end of the growing season.
3. As leaves of tall herbs are supplied by remobilised NSC of reserves, they achieve a later seasonal foliar C autonomy.

We applied a strict comparative approach by using four small-tall pairs of taxa of the same genus or families.

## Materials and methods

The study area is situated close to the Furkapass (2436 m a.s.l.) in the Swiss central Alps in the core of the alpine belt (ca. 300 m above the upper, climatic treeline). Annual precipitation at study site ranges between 1400 and 1700 mm. Strong winds in winter enlarge the variability of the annual precipitation amounts through uneven snow distribution. The growing season lasts 2.5–3.5 months, depending on local snow melt date. The mean air temperature during the warmest period (mid-June till late August) is 8–9.5 °C and the precipitation sum during the growing season (Jun-Aug) varies between 350 and 500 mm (weather station at the study site, www.alpfor.ch/weather.shtml). Freezing events can occur at any time during the growing season.

The alpine plant species that were studied (small *versus* tall herbs, Table 1) were all perennial forbs with either individual, free-standing shoots (*Ligusticum mutellina, Gnaphalium norvegicum, Gnaphalium supinum, Rumex alpestris*) or monospecific clusters of shoots (clones of *Gentiana punctata, Peucedanum ostruthium, Rumex alpinus,* named monospecific stand hereafter). All the aboveground plant organs are newly formed each season, except for *Gentiana acaulis*, a species that retains its leaves for several seasons. The study species grow across short distances (< 100 m) in four types of plant communities: both *Gentiana* species, *Ligusticum* and *Gnaphalium norvegicum* grow in the Caricion curvulae, the dominant alpine grassland type over siliceous bedrock dominated by *Carex curvula*. *P. ostruthium* and *R. alpestris* belong to tall herbfields of the Adenostylion type and *R. alpinus* is confined to locations with high soil nutrient contents due to former cattle resting (Rumicion alpini; Delarze and Gonseth 2008). *Gnaphalium supinum* is a typical species of late melting snow beds, but also extends into the Caricion curvulae grassland. The majority of the plant individuals were collected around the ALPFOR research station (46° 34′ 36′′ N, 8° 25′ 17′′ E, at SE exposure). A small number of additional samples were collected on the W-slopes of Blauberg (46° 33′ 58′′ N, 8° 24′ 51′′ E). All plant individuals were collected within 2410–2520 m a.s.l.

**Table 1:** Selected pairs of small *versus* tall plant species of the same genus or family. For *G. punctata/G. purpurea* and *L. mutellina/L. mutellinoides* a clear distinction of the species is only possible when they are flowering

Small herbs	Tall herbs	Family
*Gentiana acaulis* L.	*Gentiana punctata* L. *Gentiana purpurea* L.	Gentianaceae
*Ligusticum mutellina* (L.) Crantz *Ligusticum mutellinoides* Vill.	*Peucedanum ostruthium* (L.) W. D. J. Koch	Apiaceae
*Rumex alpestris* Jacq.	*Rumex alpinus* L.	Polygonaceae
*Gnaphalium supinum* L.	*Gnaphalium norvegicum* Gunnerus	Asteraceae

For the early phenological developmental stages 1–2, both species could have been sampled, but for the later stages it was exclusively *G. punctata* and *L. mutellina*

### Functional growth analysis

In order to study biomass production and dry matter allocation at peak biomass, four pairs of small *versus* tall plant species of the same genus or family (Table 1) were collected. However, in the *Rumex* pair, the smaller species *R. alpestris* was only slightly shorter but far more slender. In the *Gnaphalium* pair, the tall *G. norvegicum* is not very tall (ca. 15–20 cm height) compared to the other selected tall forbs, but much taller than *G. supinum*.

Six individuals per species were completely excavated at peak biomass, i.e., when plants were flowering (July/August 2013), including all rhizomes and roots (some fine roots may have been lost), washed and then the plant samples were separated into the following four major plant compartments (according to functional criteria, see Körner 1991, 1994): (1) leaves without petioles, (2) stems with petioles and inflorescences (without ripe seeds), (3) rhizome/thick roots (diameter > 4.5 mm) and (4) smaller roots (diameter: < 4.5 mm). For both *Rumex* and *G. punctata* species, the smaller roots included roots of 2–4.5 mm diameter (contrasting the thick tap roots/rhizomes) but for all other species, roots mainly consisted of small roots of < 2 mm in diameter.

For specific leaf area at peak biomass (SLA, m^2^ kg^−2^), the total leaf area, measured by a scanner (Epson Expression 1680, Epson, GER) and the free software ImageJ 1.38× (Rasband 2014), was divided by the leaf dry weight.

In addition to the harvest of individual plants, for the tall species *G. punctata*, *R. alpinus* and *P. ostruthium* that form monospecific stands, two 50 cm × 50 cm plots were harvested and completely excavated (biomass and allocation parameters per m^2^). The leaf, stem, rhizome, and root mass fractions were calculated as the percentage of the plant's total dry biomass. Leaf area ratios (LAR) were obtained by multiplying the leaf mass fraction (LMF) with the SLA. All plant samples were dried at 80 °C for 48 h and prior to weighing, re-dried and cooled in a vacuum desiccator.

### Non-structural carbohydrate (NSC) analysis

To assess the seasonal dynamics of non-structural carbohydrate concentrations plants were collected at four defined phenological developmental stages. Each species achieved these stages at different dates throughout the growing season 2013. Stage 1 was attained when the first leaf bud or leaf appeared (sampled between June-24 and July-12). At stage 2, the first leaf was fully expanded and/or the flower bud became visible (sampled between July-9 and July-18), at stage 3, the plant was flowering and aboveground biomass reached its seasonal peak (harvested between July-30 and August-16) and at stage 4, seeds became ripe and denoted as end of the growing season (sampled between Sept-2 and Sept-24).

Six individuals per species and per developmental stage were carefully dugout (for the very small *G. supinum* several individuals were pooled to get enough plant material). For the phenological stages 1, 2 and 4, only parts of belowground organs of each individual were excavated, just to have sufficient tissue amounts for the NSC analysis, but for stage 3 at peak biomass the entire plants were excavated (see functional growth analysis). Rhizomes and roots of *R. alpinus* and *G. punctata* could be visually subdivided by years, hence, the three last years of belowground organs were analysed year by year (2010, 2011 and 2012). In *P. ostruthium* thick rhizomes are commonly connected with thinner stolons, thus, the rhizome (named “first rhizome” hereafter), the interconnecting “belowground stolon” and the subsequent rhizome (named “second rhizome”) were analysed separately. All plant samples were dried at 80 °C for at least 48 h and then ground to a fine powder in a ball mill (Mixer Mill MM 400, Retsch, GER).

A modified version of the NSC analysis described in Wong (1990) and Hoch et al. (2002) was performed. Of each plant sample, 4–5 mg of the ground material were mixed with 2 ml hot, distilled water and extracted for 30 min. Two aliquots of each 200 μl were mixed with either 100 μl invertase (breaking sucrose into glucose and fructose) or fructanase (fructanase mixture, Megazyme International Ireland, IRE; splits fructan into fructose and glucose), and further, isomerase was added to convert fructose into glucose. Glucose was then converted enzymatically into gluconate-6-phosphate (hexokinase from Sigma-Aldrich, USA) and its concentration was determined photometrically in a 96-well microplate photometer (HR 7000, Hamilton, USA). The remaining extract was mixed with 500 μl dialysed clarase (α-amylase from *Aspergillus oryzae*, Enzyme Solutions Pty Ltd., AUS) and incubated overnight at 40 °C to digest starch to glucose. Again, a 200 μl aliquot was mixed with fructanase. Then, the concentration of the resultant total glucose (representing total NSC) was determined photometrically as described above. The subtraction of simple soluble sugars (glucose, fructose, and sucrose; named simple sugars hereafter) from the concentration of the fructanase mixture yielded the amount of fructan. Starch was calculated as total NSC (measured on the second day) minus fructan and simple sugars. Slightly negative starch values, presumably due to matrix effects were set to zero. As standards, we used starch-, inulin- and sucrose-solutions and orchard leaves (Leco, USA). All concentrations are expressed on a dry matter basis in mg g^−1^.

### NSC pools

By combining the NSC concentrations with the dry weights of the four plant compartments, the NSC pools at peak biomass (stage 3) were estimated for individuals (in mg per plant) as well as for monospecific stands (in g m^−2^).

### Pulse labelling with enriched δ^13^C

A pulse labelling experiment with enriched ^13^CO_2_ was carried out in the three tall species *G. punctata*, *R. alpinus* and *P. ostruthium* to explore at what phenological stage leaves become autonomous, that is, when the leaves are exporting fresh photosynthetic products to belowground organs rather than vice versa. The ^13^CO_2_ pulse was applied on the aboveground plant biomass of three individuals per species at two different phenological stages: (1) leaf was visible but not fully unfolded, (2) one leaf was at least fully expanded (ESM Fig. 2). We released enriched ^13^CO_2_ from ^13^C enriched sodium bicarbonate (400 mg 99% ^13^C; Cambridge Isotope Lab., USA; individual-A of *R. alpinus* at stage 1 was exposed to 200 mg only) in a transparent, cylindrical, acrylic glass chamber placed over the plant individuals (chamber volume = 31.4 L, equipped with five mini-ventilators and an open path infrared gas analyser; CARBOCAP® Carbon Dioxide, Probe GMP343, measuring range 0–2000 ppm CO_2_, Vaisala, FIN) by adding 40 ml of 1 N hydrochloric acid with a syringe. The reaction generated a high CO_2_ concentration (mixture of ^13^CO_2_ and ^12^CO_2_ of 838 ± 57 ppm; mean ± S.E.) and plants were exposed to it for c. 30 min. The ^13^C pulse labels were applied to the replicated individuals during morning hours (8:00–12:00) on seven consecutive days. Leaf samples were precisely taken after 0 h, 24 h, 48 h and 72 h (expressed in days in the following). After 3 days the plant individuals were completely excavated and separated into the four major plant compartments (only youngest roots were taken). Non-^13^CO_2_-enriched plans served as controls.

All plant samples were dried for 48 h at 80 °C, ground to a fine powder and analysed for δ^13^C by mass spectrometry (elemental analyzer EA-1110; Carlo Erba Thermoquest, Milan, ITA; split interface Conflo II and mass spectrometer Delta S; Thermo Finnigan Mat, Bremen, GER) at the Paul Scherrer Institute (PSI) in Villigen (Switzerland). The isotope ratio (abundance between heavy and light isotope) was expressed in the delta (*δ*) notation (in ‰; Carter and Barwick 2011).

### Statistical analysis

The statistical analysis primarily focused on the distinction between small and tall species. The SLA, LAR, biomass and pulse labelling data were analysed with nonparametric Kruskal–Wallis/Wilcoxon rank-sum tests. NSC data were power-transformed (*x*^0.3^), residual distributions were visually assessed and mixed-effects models (with the R-package nlme, Ver. 3.1–117, Pinheiro et al. 2014) for the total NSC, simple sugars, fructan, and starch concentrations (as separate dependent variables) were calculated with fixed factors: plant size (nested within plant pair), plant compartment and phenological stage, and with the random factor: plant pair. To detect the NSC differences between the compartments and the different phenological stages, for each compartment and plant species, posteriori Tukey's Honest Significant Difference (HSD) tests were performed. All statistical analysis was carried out with the software R 3.1.1 (R Core Team 2014).

## Results

Except for *G. norvegicum*, the selected tall herbs protruded the average alpine grassland canopy height by several times. Comparing the plant height between the small and tall species, the tall species were by 2.0–13.4 times taller than the small companions, with the most extreme contrast in *Gnaphalium* and the smallest difference in the *Rumex* pair (Table 2).

**Table 2:** Plant height (including flower or bud), height ratio tall to small, specific leaf area (SLA, m^2^ kg^−1^) and leaf area ratio (LAR, m^2^ kg^−1^, product of LMF and SLA) at peak biomass (phenological stage 3, mean ± S.E., *n* = number of replicates)

Species	Height (cm)		Height ratio	SLA (m^2^ kg^−1^)		LAR (m^2^ kg^−1^)		*n*
Small/tall	Small	Tall	Tall: small	Small	Tall	Small	Tall	
*G. acaulis/G. punctata*	7.3 ± 0.6^a^	25.2 ± 2.6^b^	3.5	11.1 ± 0.4^a^	18.3 ± 5.7^a^	5.3 ± 0.3^a^	2.1 ± 0.3^b^	5–6
*G. punctata* stand					13.5 ± 0.8		2.1 ± 0.7	2
*L. mutellina/P. ostruthium*	17.6 ± 1.8^a^	57.8 ± 3.3^b^	3.3	11.1 ± 1.0^a^	17.2 ± 0.4^b^	1.3 ± 0.2^a^	2.3 ± 0.4^b^	6
*P. ostruthium* stand					20.8 ± 3.3		2.4 ± 0.2	2
*R. alpestris/R. alpinus*	38.6 ± 0.9^a^	78.3 ± 5.2^b^	2.0	28.5 ± 1.1^a^	18.7 ± 1.5^b^	2.1 ± 0.2^a^	1.9 ± 0.2^a^	6
*R. alpinus* stand					19.4 ± 0.3		1.1 ± 0.1	2
*G. supinum/G. norvegicum*	1.1 ± 0.1^a^	15.0 ± 0.9^b^	13.4	23.9 ± 2.5^a^	26.2 ± 7.9^a^	7.3 ± 0.7^a^	7.2 ± 2.0^a^	6

Three out of four tall species form monospecific stands (additional SLA, LAR values). Different letters indicate significant differences within pairs at *p* < 0.05 (Wilcoxon rank-sum test)

### Specific leaf area (SLA)

The mean specific leaf area (SLA) across all eight species was 19.4 ± 1.4 m^2^ kg^−1^ (± S.E.), values typically reported for alpine species. However, a pronounced difference in SLA between small *versus* tall pairs was not observed. The SLA was higher in the tall herb of the *Ligusticum /Peucedanum* pair (*p* = 0.002), but in the *Rumex* pair it was the other way round (Table 2; *p* = 0.002). Species of *Gnaphalium* had similar SLA values irrespective of the stature.

### Biomass allocation

**Fig. 1:**
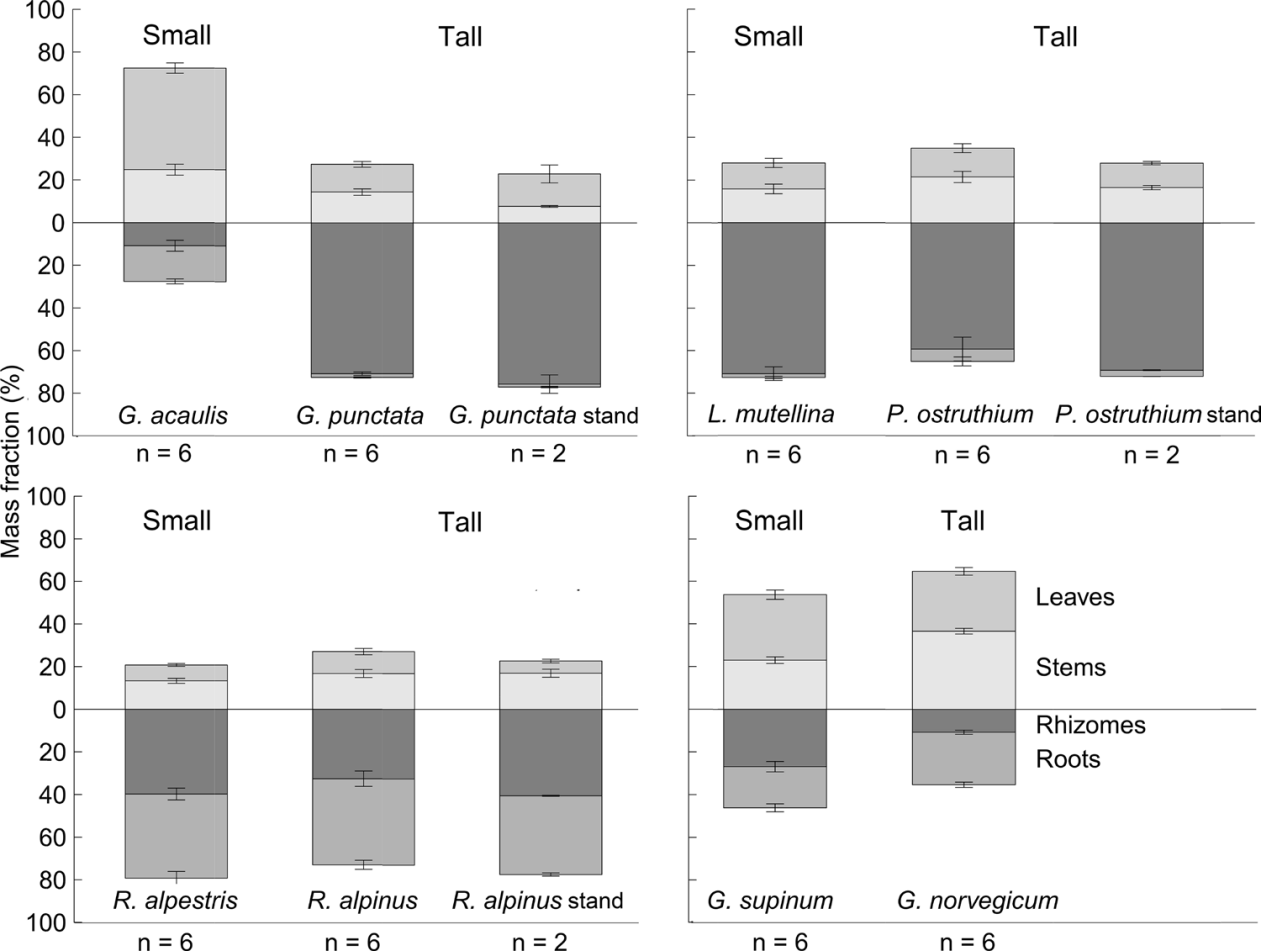
Comparison of mass fractions (% of dry biomass) ± S.E. of the four small *versus* tall pairs; “stand” means monospecific stands of each of the three tall species (mean ± S.E., 50 × 50 cm)

For the biomass allocation, the two different harvesting methods, namely the selection of individuals *versus* dense monospecific stands for three of the tall species yielded very similar mass fractions. The absolute aboveground dry matter was obviously always higher in the tall species compared to the small ones (Table 3; Wilcoxon *W* = 86, *p* < 0.001). A preferential allocation to aboveground parts was detected in *G. acaulis* (small herb with evergreen foliage) and both *Gnaphalium* species. In the five other species, the allocation to belowground biomass dominated, irrespective of the small-tall stature, thus not supporting our hypothesis 1 (Fig. 1, Table 3). Across all species, the rhizome mass fraction (40.2 ± 3.5%; mean ± S.E. without the dense stand harvest data) was substantially higher than the root mass fraction (19.5 ± 2.2%) except for both *Rumex* species, with equally high root and rhizome mass fractions, and for *G. acaulis* and *G. supinum* with higher root fractions*.* The stem mass fraction (20.8 ± 1.2%) was similar to that of leaves (20.3 ± 2.0%). The small *G. acaulis* with its overwintering leaves exhibited the highest leaf mass fraction of all species (c. 48%)*.* Within pairs, biomass allocation differed substantially in the *Gentiana* and *Gnaphalium* pairs. The rhizome mass fraction of the tall *Gentiana* was over six times higher than in the small *Gentiana* and in the small *Gnaphalium* it was over twice as high as in the tall *Gnaphalium*. We expected a larger stem mass fraction in tall species; however, a statistically higher stem fraction was only observed in the tall *Gnaphalium* species (Fig. 1, Table 3).

**Table 3:** Mean (± S.E.) dry matter (g) and mass fractions (%) of small *vs.* tall species and monospecific stands of three tall herbs at peak biomass (stage 3)

Species	Compartment	Dry matter (g)		Dry matter (g m^−2^)	Mass fraction (%)			
Small/tall		Small	Tall	Tall stand	Small	Tall	Tall stand	*p* value
*G. acaulis/*	Leaf	0.26 ± 0.04	1.42 ± 0.35	179.9 ± 11.5	47.6 ± 2.4^a^	13.0 ± 1.3^b^	15.2 ± 4.2^b^	0.008
*G. punctata*	Stem	0.13 ± 0.02	1.36 ± 0.09	96.0 ± 15.2	24.8 ± 2.5^a^	14.5 ± 1.5^b^	7.7 ± 0.5^b^	0.011
	Rhizome	0.06 ± 0.01	7.22 ± 1.14	964.1 ± 260.1	10.8 ± 2.6^a^	70.8 ± 0.8^b^	75.7 ± 4.3^b^	0.006
	Root	0.09 ± 0.01	0.19 ± 0.05	18.7 ± 8.0	16.7 ± 1.2^a^	1.7 ± 0.3^b^	1.4 ± 0.3^b^	0.008
	Aboveground	0.39 ± 0.05	2.78 ± 0.42	276.0 ± 3.8	72.4 ± 3.1^a^	27.5 ± 0.8^b^	22.9 ± 4.7^b^	0.007
	Belowground	0.15 ± 0.02	7.40 ± 1.19	982.8 ± 268.1	27.6 ± 3.1^a^	72.5 ± 0.8^b^	77.1 ± 4.7^b^	0.007
*L. mutellina/*	Leaf	0.17 ± 0.05	3.71 ± 1.09	117.4 ± 7.7	12.2 ± 2.2	13.5 ± 2.1	11.4 ± 0.8	0.842
*P. ostruthium*	Stem	0.18 ± 0.02	4.94 ± 0.96	173.2 ± 33.5	15.8 ± 2.3	21.5 ± 2.6	16.6 ± 1.0	0.279
	Rhizome	0.97 ± 0.22	15.67 ± 5.23	715.4 ± 94.3	70.9 ± 3.2	59.2 ± 5.6	69.1 ± 0.2	0.152
	Root	0.03 ± 0.01	1.60 ± 0.65	29.8 ± 3.9	1.8 ± 0.6	5.8 ± 2.1	2.9 ± 0.0	0.433
	Aboveground	0.35 ± 0.06	8.65 ± 2.00	290.6 ± 41.2	28.0 ± 3.1	35.0 ± 3.7	28.0 ± 0.2	0.237
	Belowground	0.99 ± 0.22	17.28 ± 5.59	745.3 ± 98.2	72.0 ± 3.1	65.0 ± 3.7	72.0 ± 0.2	0.237
*R. alpestris/*	Leaf	0.30 ± 0.07	8.705 ± 1.70	168.9 ± 1.9	7.4 ± 0.7	10.3 ± 1.5	5.7 ± 0.8	0.074
*R. alpinus*	Stem	0.48 ± 0.08	13.69 ± 1.78	519.3 ± 135.7	13.3 ± 1.1	16.8 ± 1.9	16.9 ± 1.9	0.296
	Rhizome	1.65 ± 0.46	28.90 ± 7.86	1220.3 ± 181.9	39.8 ± 2.7	32.6 ± 3.6	40.5 ± 0.3	0.130
	Root	1.55 ± 0.43	34.39 ± 6.67	1109.6 ± 149.5	39.5 ± 3.2	40.4 ± 2.2	36.9 ± 0.8	0.416
	Aboveground	0.78 ± 0.14	22.40 ± 3.39	688.2 ± 137.6	20.8 ± 1.5	27.0 ± 3.2	22.6 ± 1.0	0.344
	Belowground	3.21 ± 0.84	63.29 ± 14.12	2329.9 ± 331.4	79.2 ± 1.5	73.0 ± 3.2	77.4 ± 1.0	0.344
*G. supinum/*	Leaf	0.04 ± 0.01	0.12 ± 0.01		30.8 ± 2.2	28.0 ± 1.8		0.394
*G. norvegicum*	Stem	0.03 ± 0.01	0.16 ± 0.02		23.0 ± 1.5	36.7 ± 1.4		0.002
	Rhizome	0.03 ± 0.01	0.05 ± 0.01		26.9 ± 2.4	10.7 ± 1.0		0.002
	Root	0.02 ± 0.00	0.10 ± 0.01		19.3 ± 1.9	24.6 ± 1.3		0.065
	Aboveground	0.07 ± 0.01	0.28 ± 0.03		53.7 ± 2.1	64.7 ± 1.2		0.004
	Belowground	0.06 ± 0.01	0.15 ± 0.01		46.3 ± 2.1	35.3 ± 1.2		0.004

Differences between mass fractions within each pair are indicated by *p* values and different letters (*p* < 0.05)

### Leaf area ratio (LAR, product of LMF and SLA)

We anticipated a lower LAR in the small herbs. Across the eight species, the LAR was 3.7 ± 0.4 m^2^ kg^−1^ and did not differ between small *versus* tall plants (Table 2). In the two pairs with significant differences, the LAR of the small *L. mutellina* was lower than the LAR of tall *P. ostruthium* (*p* = 0.026), but in the *Gentiana* pair, the opposite was the case with over twice as high LAR in the small *G. acaulis* (*p* = 0.015), most likely because of its high LMF.

### Non-structural carbohydrates (NSC)

**Fig. 2:**
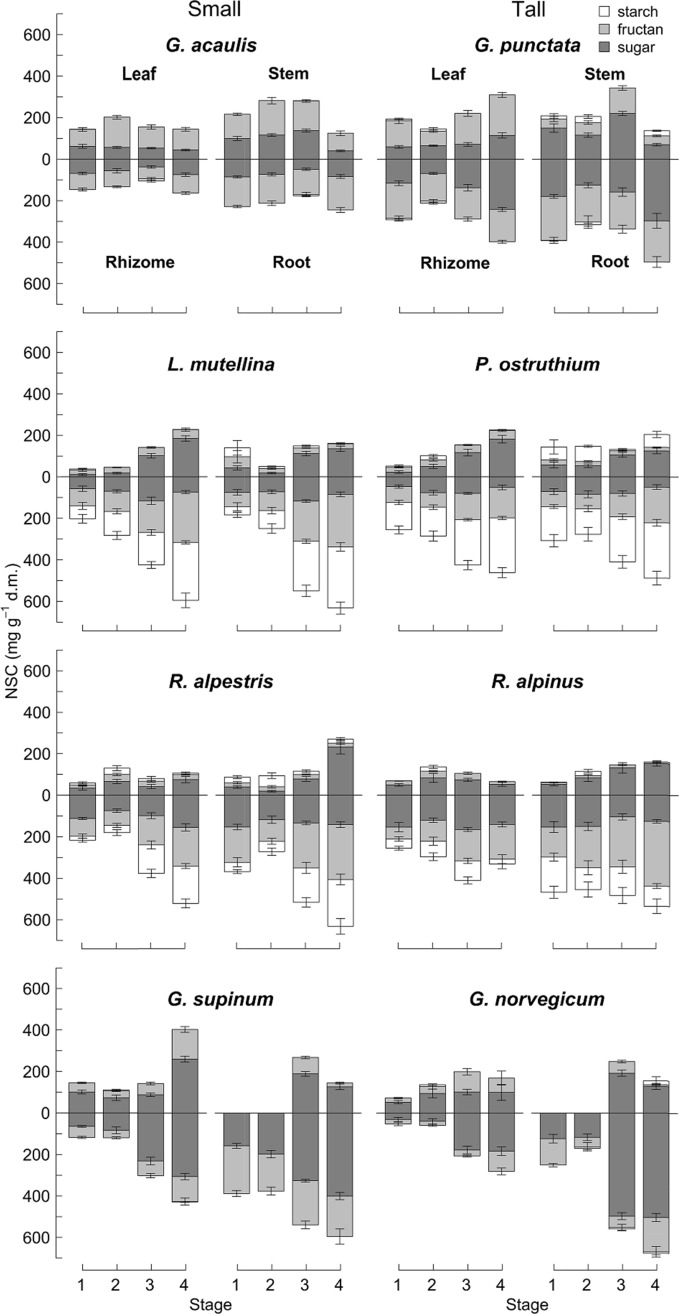
Non-structural carbohydrate (NSC) concentrations (mean ± S.E.). NSC consisted of simple sugars, fructan and starch (mg g^−1^ d. m.) of the four small *versus* tall pairs in different compartments at four phenological stages (*n* = 4–6). No stems in the *Gnaphalium* pair were available during the first two stages

Generally, and independently of the stature of the species, belowground organs (rhizomes and roots) showed the highest NSC concentrations, whereas NSC concentrations were typically lower in leaves and stems (Fig. 2). Across all species and all phenological stages, absolute NSC concentrations in the rhizomes amounted to 273 ± 10 mg g^−1^ (d. m.; mean ± S.E.), thus, dry matter consisted of 27% of NSC. The roots had a mean NSC concentration of 367 ± 14 mg g^−1^ and leaves contained 144 ± 6 mg g^−1^ NSC. Fructans contributed significantly to NSC, were present in all species as well as in all biomass compartments, and also contributed to the seasonal NSC dynamics. Starch concentrations were particularly high in roots and rhizomes of the *Ligusticum*/*Peucedanum* and the *Rumex* pair, but nearly absent in the different compartments of both, the *Gentiana* and *Gnaphalium* pairs (Fig. 2).

The mixed-effect models analysis across all four pairs and biomass compartments showed that total NSC concentrations did not differ between species of small *versus* tall stature (Table 4), and this was also the case, when the individual NSC components were tested (Table 4). Thus, tall stature species did not differ from low stature species with respect to their NSC concentrations.

**Table 4:** The effect of plant stature (size), plant compartment and phenological stages on total non-structural carbohydrates (NSC), simple free sugars, fructan and starch concentrations

Dependent variable	*d.f*	*F*	*p *values
Total NSC			
(Size/pair)	1, 3	0.5	0.532
Compartment	3, 689	169.2	< 0.001
Stage	3, 689	69.6	< 0.001
Compartment x stage	9, 689	4.3	< 0.001
Simple sugars			
(Size/pair)	1, 3	0.3	0.630
Compartment	3, 691	39.0	< 0.001
Stage	3, 691	50.0	< 0.001
Size x stage	3, 691	2.9	< 0.001
Compartment x stage	9, 691	2.0	< 0.001
Fructan			
(Size/pair)	1, 3	1.0	0.387
Compartment	3, 687	200.9	< 0.001
Stage	3, 687	16.2	< 0.001
Size x compartment	3, 687	9.8	< 0.001
Compartment x stage	9, 687	9.5	< 0.001
Starch			
(Size/pair)	1, 3	0.4	0.558
Compartment	3, 683	65.8	< 0.001
Stage	3, 683	4.0	0.008
Size x stage	3, 683	8.2	< 0.001
Compartment x stage	9, 683	5.6	< 0.001

Small and tall species were “nested” under pair. Only compartments present in all species were included in the statistical analysis (balanced design). Models were recalculated after removing non-significant interactions terms from the models

Total NSC concentrations largely differed between different compartments (highest *F* values) and phenological stages (slightly lower *F* values) more strongly than between species. Total NSC concentrations generally increased over the four phenological stages, but not to the same extent in the different plant compartments (see statistically significant compartment x stage interaction, Table 4). For instance, the NSC concentration in rhizomes and roots slightly decreased from stage 1 to stage 2 in some of the species, which indicates a remobilisation of stored NSC for early season growth, and also the stem NSC concentration decreased from stage 3 to stage 4 (end of the season).

*Simple sugar* concentrations were higher in roots than rhizomes, followed by stems and leaves. Simple sugar concentrations changed with phenology and plant stature (stature x stage interaction; Table 4, Fig. 2). At stage 3, simple sugar concentrations in stems were higher in the tall compared to the small species. Highest *fructan* concentrations were measured in roots followed by rhizomes. The small species had relatively higher fructan concentrations in roots and stems than the tall species (significant stature x compartment interaction). In those species that stored *starch*, concentrations were typically higher in belowground organs than in aboveground organs. Starch concentrations were lower in taller plants at stage 3 (stature × stage interaction). Because the mixed effect model across all four pairs may not mirror changes in NSC concentrations within a single plant pair, we add a few results for each pair separately.

#### *Gentiana* pair

The total NSC concentrations in the rhizomes and roots were higher in the tall species *G. punctata* than in the small *G. acaulis* species (Fig. 2), supporting our hypothesis 2. Interestingly, differently aged rhizome segments (2010–2012) of the tall *Gentiana* showed very similar seasonal variation in NSC concentrations. Thus, several year-old rhizomes still represent active storage organs in this species, also supported by the observation that rhizome segments older than five years in a 12-year-old rhizome of *G. punctata* stored even more NSC than younger segments due to higher concentrations of simple sugars (data not shown).

#### *Ligusticum*/*Peucedanum* pair

Both species showed a rather similar and steady NSC increase over the season, related to higher starch concentrations (Fig. 2). Foliar NSC concentrations were also rising over the growing season, mainly through an increase in simple sugars at stages 3 and 4.

#### *Rumex* pair

The tall *Rumex* species showed stable NSC concentrations in the rhizomes (analysed separately for 2010, 2011, 2012) at the beginning of the season, followed by an increase in NSC at peak biomass and a slight decrease at stage 4. Roots emerging from a given rhizome segment had always higher NSC concentrations across the four stages than the rhizome segments. In contrast to the above-mentioned 12-year-old *Gentiana punctata* rhizome, a 7-year-old rhizome of a *Rumex alpinus* individual revealed decreasing total NSC concentrations with increasing age, and the oldest rhizome segment contained mainly simple sugars with very little fructan and starch (data not shown).

#### *Gnaphalium﻿* pair

Both *Gnaphalium* species displayed a similar seasonal course in NSC, and fructan and simple free sugars were the dominating NSC compounds (Fig. 2). In the small *G. supinum*, the leaf NSC concentrations were constant from stage 1–3, but nearly tripled towards stage 4, exceeding leaf NSC of all other species investigated, with a mean of 403 ± 11 mg g^−1^.

### NSC pools

Irrespective of the plant stature, the belowground NSC pools were constantly larger than the aboveground NSC pools, except for the small but evergreen *G. acaulis* with a higher aboveground NSC pool (Table 5). Because of the higher overall dry matter per tall individual, NSC pools of different compartments were always larger in the tall than in the small species. However, small species always showed larger relative differences in the NSC pools between above- and belowground. For instance, in the 'small' *R. alpestris* the belowground pool was 17 times higher than the aboveground pool, whereas for the tall *Rumex alpinus* the belowground pool was 10 times bigger than the aboveground pool. In small species, the root NSC pool was larger than the rhizome NSC pool in three out of four small species. In the tall species *G. punctata* and *P. ostruthium*, the rhizome NSC pool surpassed root NSC pool substantially. In *R. alpinus*, with extremely high NSC pools in both root and rhizome, the root NSC pool was slightly larger than the rhizome NSC pool.

**Table 5:** Mean NSC pools (± S.E., mg plant^−1^ and g m^−2^) in the different plant compartments of the four small *vs.* tall pairs and in monospecific stands of three tall species at peak biomass (stage 3)

Species	Compartment	NSC pool (mg plant^−1^)		NSC pool (g m^−2^)	Ratio below-: aboveground NSC pool		
Small/tall		Small	Tall	Tall stand	Small	Tall	Tall stand
*G. acaulis /*	Leaf	40 ± 7	313 ± 83	40 ± 7			
*G. punctata*	Stem	37 ± 5	466 ± 38	33 ± 6			
	Rhizome	6 ± 1	2415 ± 426	323 ± 97			
	Root	16 ± 2	63 ± 18	6 ± 3			
	Aboveground	77 ± 6	779 ± 65	73 ± 9			
	Belowground	22 ± 2	2478 ± 302	329 ± 97	0.3	3.2	4.5
*L. mutellina/*	Leaf	24 ± 7	572 ± 177	18 ± 3			
*P. ostruthium*	Stem	27 ± 3	650 ± 139	23 ± 6			
	Rhizome	470 ± 111	6372 ± 2203	291 ± 60			
	Root	12 ± 5	635 ± 266	12 ± 3			
	Aboveground	51 ± 6	1222 ± 159	41 ± 6			
	Belowground	483 ± 79	7007 ± 1569	303 ± 60	9.5	5.7	7.4
*R. alpestris /*	Leaf	26 ± 7	917 ± 194	18 ± 2			
*R. alpinus*	Stem	56 ± 13	2003 ± 417	76 ± 29			
	Rhizome	623 ± 184	12480 ± 3490	527 ± 99			
	Root	802 ± 226	18848 ± 4164	608 ± 138			
	Aboveground	82 ± 11	2920 ± 325	94 ± 29			
	Belowground	1426 ± 206	31328 ± 3842	1135 ± 170	17.4	10.7	12.1
*G. supinum/*	Leaf	5 ± 1	24 ± 4				
*G. norvegicum*	Stem	8 ± 1	39 ± 5				
	Rhizome	10 ± 2	9 ± 1				
	Root	12 ± 1	58 ± 3				
	Aboveground	13 ± 1	63 ± 5				
	Belowground	22 ± 2	68 ± 2		1.7	1.1	

### Pulse labelling with enriched δ^13^C

**Fig. 3:**
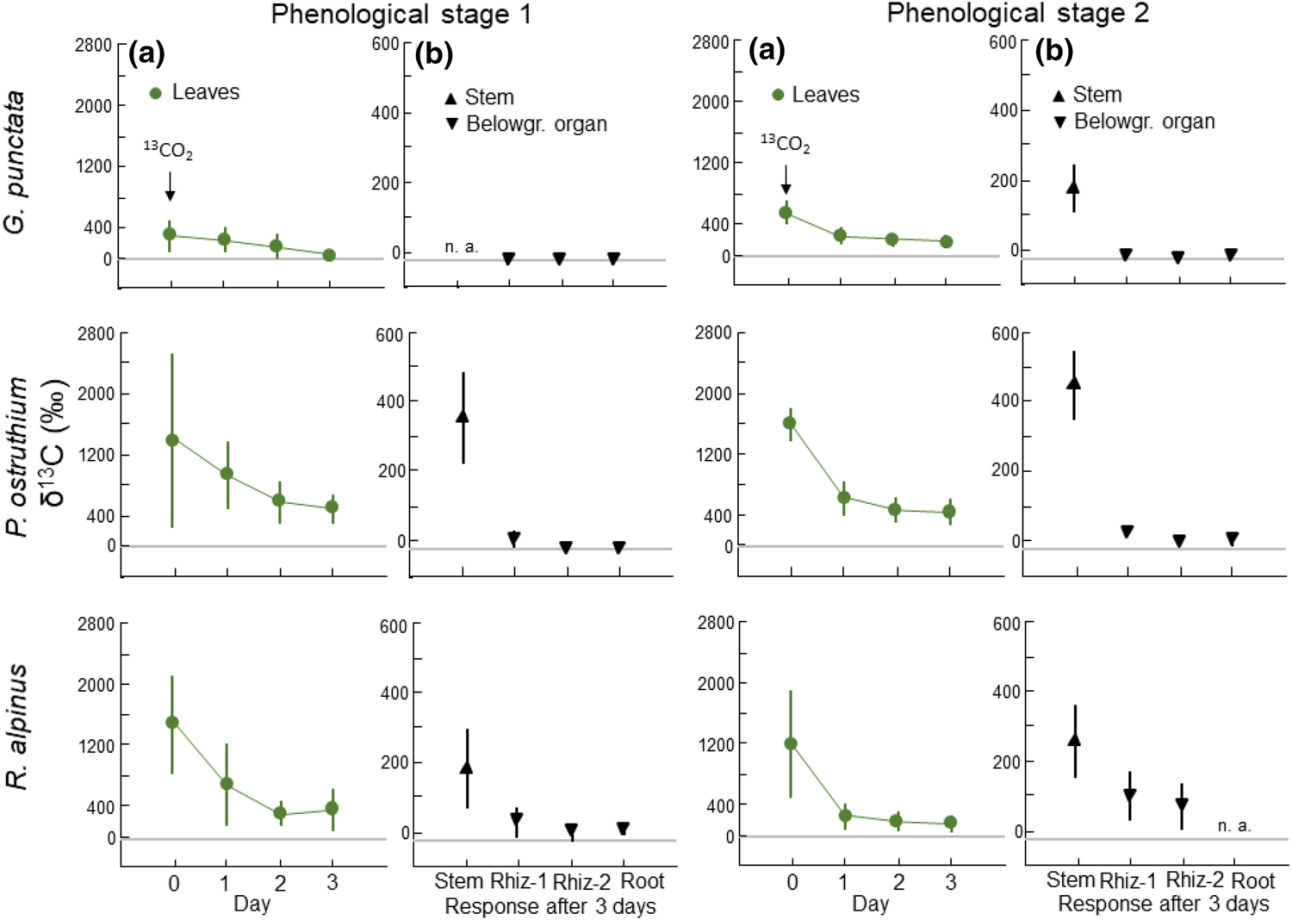
Mean δ^13^C values (‰, ± S.E.) of ^13^CO_2_ pulsed three tall herbs in **a** leaves after 0, 1, 2 and 3 days and in **b** stems, rhizomes (separated in 2012 = rhiz-1 and 2011 = rhiz-2) and youngest roots after 3 days at phenological stage 1 and stage 2. *Y* axis differs in **a** and **b**. Gray line indicates δ^13^C value in non-enriched ^13^CO_2_ control plants (range of −28.4 to −24.1‰). Not all tissues were available (n. a.)

All leaves of the plant individuals exposed to a ^13^CO_2_ pulse had clearly higher δ^13^C values (with maximum values of 2352‰) than the control plants (foliar δ^13^C of −25.7 ± 0.14‰, mean ± S.E.) right after pulsing (Fig. 3). Among the three tall species (*Gentiana, Peucedanum* and *Rumex*) and within each species, the δ^13^C values in leaves varied immensely, by over five-fold (ESM Table 1). However, leaf uptake was generally lower in *G. punctata* than in the other two species, despite similar CO_2_ concentrations (ESM Fig. 1). Leaf δ^13^C values in the three species decreased exponentially with time and after three days, leaf signals ranged between 15 and 780‰ (Fig. 3). In stems, the δ^13^C values were 267 ± 70‰ in the early stage 1 and 294 ± 53‰ in stage 2. Even at the phenological stage 1, when leaves just emerged (ESM Fig. 2), δ^13^C values were higher in belowground organs, especially in the younger rhizomes than in controls: 2.3 ± 13‰ in young rhizomes, −16 ± 6‰ in older rhizomes, and −14 ± 6‰ in roots (means ± S.E.; −26.8 ± 0.2‰ in control rhizomes, Fig. 3). *G. punctata* showed very weak ^13^C signals in the belowground organs at stage 1, in contrast to *P. ostruthium* and *R. alpinus* with higher δ^13^C values in rhizome and roots, underpinning a very early leaf autonomy in these two species, thus falsifying our hypothesis 3. At phenological stage 2, a higher fraction of the applied ^13^C pulse was translocated to rhizomes and roots, resulting in higher δ^13^C values than at stage 1 (34 ± 23‰ in younger and 13 ± 20‰ in older rhizomes, respectively and −11 ± 8‰ in roots). Again, the ^13^C signals in the *G. punctata* were weaker at stage 2 than in the other two species.

## Discussion

The heights of the tall species examined surpassed the mean height of the surrounding typical alpine vegetation. Thus, the protruding plant parts of the tall species are assumed to be exposed to climatic conditions closer to the free atmosphere than their small stature relatives. Tall plants may experience cooler day temperatures, however, their aerodynamic coupling to the free atmosphere causes them to experience warmer night temperatures. On the other hand, aerodynamically decoupled small alpine plants heat up during bright days but may cool below air temperature in clear nights due to heat loss by outgoing thermal radiation (Körner and Hiltbrunner 2018; Monteith and Unsworth 2013). There are additional caveats of the tall- cool and small-warm ‘rule’. In many tall plants, the majority of the foliage is forming a basal rosette with its own microclimate and with only the upper part of the flowering stalk and associated foliage protruding. However, this is not the case for *Rumex alpinus* and *Peucedanum ostruthium* where big leaves build a closed canopy layer with high boundary layer resistance.

Dry matter investments per foliage area (or its reverse, SLA) and the foliage produced per total plant biomass are both considered key drivers of plant growth (Poorter et al. 1990, 2015; van der Werf et al. 1998). Because LAR reflects the combination of leaf mass fraction (LMF) and SLA, and SLA was found to be the main driver of LAR (Poorter and Garnier 2007), the tall herbs with their fast growth in spring were expected to exhibit a larger SLA. However, this is not what we observed. The observed SLA across all species corresponds well with the mean SLA of 19 m^2^ kg^−1^ for alpine herbs at ca. 3000 m a.s.l. in the eastern Alps (Körner et al. 1989), and there was no small/tall distinction. The mean LAR across all species of 3.7 ± 0.4 m^2^ kg^−1^ (± S.E.) was slightly lower than the mean LAR of 4.5 m^2^ kg^−1^ across 27 small alpine herbs reported by Körner and Renhardt (1987), again without evident small/ tall differentiation. The three species with a higher LAR (5–8.2 m^2^ kg^−1^) than the overall mean: *G. acaulis* and both *Gnaphalium* species were also the species which preferentially allocated biomass to aboveground organs. Hence, neither SLA nor LAR permits any obvious distinction related to plant stature.

### Biomass allocation

Given that biomass allocation did not differ consistently between the small *versus* tall species, our hypothesis 1 that tall plants need relatively bigger belowground organs to permit a rapid growth after snowmelt was not confirmed. Körner and Renhardt (1987) reported an overall lower mean rhizome fraction (28.0 ± 3.8% for 'storage' organs), but a higher fine root fraction (30.2 ± 3.4%) for 27 small herbaceous alpine species than what we observed across the eight species, irrespective of their stature. Our small species had a mean leaf mass fraction of 24.5 ± 3.5%, very similar to the mean of 23.8 ± 2.0% of other small alpine species (Körner and Renhardt 1987), while the mean LMF for the tall species was 16.2 ± 1.7%, what we attribute to the higher stem mass fraction in the tall species. Thus, in line with the LAR data, the tall herbs actually invest relatively less in leaf biomass than the small species.

### Non-structural carbohydrates (NSC)

When all small *versus* tall plant pairs, the different biomass compartments, and the four different developmental stages were considered (in one common analysis model), the studied tall stature plants did not significantly differ from low stature species with respect to their NSC concentrations and this was also valid when simple sugar, fructan, and starch concentrations were tested separately. The higher simple sugar concentrations in stems of tall compared to small species at peak biomass may be associated with a higher phloem sap fraction, the transitory nature of stem pools (Mooney 1972).

The rise of NSC concentrations from stage 2 to 4 indicates that NSC mobilised for initial growth was rapidly restored, with foliage exporting carbon from a very early stage on (Mooney and Billings 1960; Fonda and Bliss 1966). The expected decrease in the rhizome and/ or root NSC concentrations at the beginning of the growing season (that is between stage 1 and stage 2) was only observed in rhizomes of tall *G. punctata,* and in roots of small *R. alpestris* and of tall *G. norvegicum*. The pulsing experiment with ^13^CO_2_ revealed a “later” or commonly weaker leaf autonomy in the tall *G. punctata,* compared to the very early leaf autonomy in *P. ostruthium* and *R. alpinus.* And in these two species*,* the NSC concentrations in the belowground organs remained constant between stage 1 and stage 2, hence, there was no obvious remobilization of NSC from these organs, thus, not supporting our hypotheses 2 and 3.

The highest NSC concentrations across all different organs were found in the roots with up to 700 mg g^−1^ and also the highest fructan concentrations (up to 400 mg g^−1^) were observed in the roots. Very similar values were reported for roots of Australian alpine plant species with NSC concentrations up to 620 mg g^−1^ and fructan concentrations up to 430 mg g^−1^ (Tolsma et al. 2007). Fructans are stored in the vacuole of the plant cells and enable the plant to store higher NSC concentrations (Chatterton et al. 1989; Pollock and Cairns 1991). It is known that fructan synthesis is still active at low temperatures (0–5 °C), at these temperatures plant growth is known to be more constrained than photosynthesis (Farrar 1988; Körner 2021). The high concentration of fructan across all studied species and organs calls for a routine inclusion of fructans in any NSC analysis of alpine species. Here, in our small-tall comparison, small species had higher fructan concentrations in their roots than the tall species (with a lower root mass fraction).

The data on NSC seasonal dynamics may contribute to the debate about plant priorities for either storage or growth. Chapin et al. (1990) distinguished between reserve formation (competing with growth) and accumulation of storage substances. In the *Ligusticum / Peucedanum* pair, the NSC concentrations in rhizomes started to increase already from stage 1 on, meaning that even when the demand for NSC in aboveground organs was substantial, storage of NSC occurred (in addition of the early leaf autonomy in *Peucedanum*). Nevertheless, the analysis of NSC concentrations within a specific organ does not allow differentiating between existing and newly formed carbohydrates, thus, organs with high NSC concentrations may still have high turnover rates. On the other hand, NSC concentrations in the tall *R. alpinus* and *G. punctata* individuals in which we studied rhizome age effects, indicate high NSC “deposits” in old, belowground parts that do not seem to contribute to seasonal turnover. High NSC concentrations in old(er) rhizome segments may insure plants against losses of a substantial parts of their biomass (e.g., resilience under strong herbivore pressure).

In previous experiments with removal of aboveground parts, simulating damage or mowing, hence, a minor tissue loss only, the NSC concentrations in roots or rhizomes were either not affected (Latzel et al. 2013), increased (Janeček and Klimešová 2014) or slightly decreased, though not affecting the overall survival of the plant (Kleijn et al. 2005). In a pot experiment with four meadow plants, the removal of either aboveground tissues or roots caused a decrease in NSC concentrations and NSC pools in both rhizome and roots (Janeček and Klimešová 2014). Yet, in most studies with shoot removal, belowground NSC concentrations remained stable or even increased, but so far, too few studies have been carried out on damages of belowground organs and their effects. Marmots and small rodents are known to largely feed on roots and rhizomes; thus, it is rather surprising that these relationships between rhizome damages, NSC concentrations, and plant resilience have not been studied in detail. Aryal et al. (2015) reported a positive correlation between the sum of dry matter protein, carbohydrate, and fat concentrations in plants and the consumption by the Himalayan marmot, indicating selective feeding of this herbivore.

Our seasonal harvests under common alpine life conditions revealed little influence of aboveground growth -irrespective of the plant stature- on NSC concentrations in the belowground organs, so, NSC storage has an overwhelming priority for these alpine plants. Moreover, CO_2_ enrichment and shading experiments in situ revealed that carbon is not a limiting resource for alpine plants (Körner et al. 1997; Inauen et al. 2012; Möhl et al. 2020) and tall alpine forbs are no exception in this respect.

NSC pools size (estimated at peak biomass only) scaled symmetrically with biomass. It seems, however, that the rhizome NSC pools played a greater role in the tall stature species, whereas in the small species, roots represent the bigger NSC pool. Tall species also had a higher aboveground NSC pool than the small species because of the bigger stems. The relative pool sizes between above- *versus* belowground compartments displayed lower relative NSC differences in tall compared to small species. It is hard to attribute a functional significance to these relative differences. It may well be that the tall herbs simply need a strong mechanical (rhizomatous) basis for their tall shoots. In case of heavy damage, sprouts can rise directly from rhizomes, so, a network of rhizomes also contributes to resilience (Klimeš 1992; Klimešová et al. 2018a, b). The high NSC concentrations in rhizomes of tall species may contribute to tissue renewal during the remaining, short growing season. Thus, it depends on the degree and timing of disturbances whether stores become reserves in tall forbs.

Tracking ^13^C pulse labelling signals from leaves to belowground organs helped identifying the direction and rate of C transfer during early growth. It is well established that leaves gain C autonomy, and thus, become net C exporters very early during their expansion. For the majority of dicotyledonous plants, the transition between cessation of import and start of export of NSC seems to occur when leaves are 30–60% fully expanded. Import of NSC has been found to continue for a certain period, even when the export of NSC has already started (Turgeon 1989). Referring to the tall species, we expected a pronounced reserve mobilisation from the rhizomes and therefore a late achievement of C autonomy of the leaves. We found only in *G. punctata* an early seasonal decrease in the NSC concentrations of belowground organs and a later C autonomy in leaves.

## Conclusions

The study of biomass allocation at peak biomass and NSC storage over a growing season in congeneric or closely related pairs of typically small *versus* exceptionally tall alpine herbs revealed that tall species are simply bigger and are not characterised by special biomass investments. Surprisingly, there was also no overwhelming difference in the production and use of NSC over the growing season between the tall and the small species, except for higher simple sugar concentrations in stems of tall species at biomass peak, and an overall preferential allocation of NSC to belowground organs was observed. The ^13^C pulse labelling in tall species showed that the allocation of newly assimilated C to belowground organs has a high priority already from early developmental stages on, and independently of the already massive NSC stores.

Conclusively, our findings do not place tall alpine herbs in a special category in terms of biomass investment and carbohydrate storage. Growing tall is not simply related to greater belowground NSC stores. Tall species even revealed lower belowground/ aboveground ratios of their NSC pools. Thus, the means by which these tall herbs 'break the rule' seem to partly rely on the mechanical strength of these massive rhizomes. Without severe disturbances, rhizome parts may reach substantial age (> 10 years), fully packed with carbohydrates and contributing to the persistence of these plant species. We do not see a consistent association of tall taxa with nutrient-rich locations as was suggested by Steffen et al. (2016). While some of the studied tall herbs are clearly associated with fertile microhabitats (e.g., *Rumex alpinus),* others (e.g., *Gentiana punctata*) are clearly not. Tall alpine herbs are not facing any obvious disadvantage in their carbon relations compared to their smaller stature relatives. We infer that NSC stores are not a limiting factor for growth in the tall alpine species.

## Supplementary Information

Below is the link to the electronic supplementary material.

Supplementary file1 (PDF 779 kb)
