# Supplementary material for: Biomass allocation and seasonal non-structural carbohydrate dynamics do not explain the success of tall forbs in short alpine grassland
Source: Oecologia. 2021 May 28;197(4):1063–77. doi: 10.1007/s00442-021-04950-7 (PMC8591020; doi:10.1007/s00442-021-04950-7)

Electronic Supplementary Material Hiltbrunner et al.

ESM Table 1: Raw data of $\delta^{13}C$ values in three tall forbs after $^{13}CO_2$ pulsing, ordered by species, phenostage , days and tissues (Ge_pu: *Gentiana punctata*, Pe_os: *Peucedanum ostruthium*, Ru_alpi: *Rumex alpinus*)

NAME	INDIV	DAY	TISSUE	PHENO-STAGE	δ13C	CONTROL δ13C
P_Ge_pu_A1_leaf0d	A1	0	leaf	1	120.25	-27.042
P_Ge_pu_A1_leaf1d	A1	1	leaf	1	179.82	-27.042
P_Ge_pu_A1_leaf2d	A1	2	leaf	1	36.43	-27.042
P_Ge_pu_A1_leaf3d	A1	3	leaf	1	39.42	-27.042
P_Ge_pu_A1_stem	A1	3	stem	1	.	.
P_Ge_pu_A1_rhiz_1	A1	3	rhiz	1	-25.52	-26.530
P_Ge_pu_A1_rhiz_2	A1	3	rhiz	1	-26.84	-26.841
P_Ge_pu_A1_root_3	A1	3	root	1	-24.93	-26.650
P_Ge_pu_B1_leaf0d	B1	0	leaf	1	576.84	-27.042
P_Ge_pu_B1_leaf1d	B1	1	leaf	1	469.03	-27.042
P_Ge_pu_B1_leaf2d	B1	2	leaf	1	378.94	-27.042
P_Ge_pu_B1_leaf3d	B1	3	leaf	1	74.26	-27.042
P_Ge_pu_B1_stem	B1	3	stem	1	.	.
P_Ge_pu_B1_ rhiz_1	B1	3	rhiz	1	-24.95	-26.530
P_Ge_pu_B1_ rhiz_2	B1	3	root	1	-25.36	-26.391
P_Ge_pu_B1_root_2	B1	3	root	1	-25.19	-26.650
P_Ge_pu_C1_leaf0d	C1	0	leaf	1	133.86	-27.042
P_Ge_pu_C1_leaf1d	C1	1	leaf	1	40.15	-27.042
P_Ge_pu_C1_leaf2d	C1	2	leaf	1	11.24	-27.042
P_Ge_pu_C1_leaf3d	C1	3	leaf	1	-9.28	-27.042
P_Ge_pu_C1_stem	C1	3	stem	1	.	.
P_Ge_pu_C1_ rhiz_1	C1	3	rhiz	1	-26.01	-26.530
P_Ge_pu_C1_ rhiz_2	C1	3	rhiz	1	-26.09	-26.391
P_Ge_pu_C1_ root_6	C1	3	root	1	-26.13	-26.650
P_Ge_pu_A2_leaf0d	A2	0	leaf	2	398.43	-26.394
P_Ge_pu_A2_leaf1d	A2	1	leaf	2	231.4	-26.394
P_Ge_pu_A2_leaf2d	A2	2	leaf	2	168.5	-26.394
P_Ge_pu_A2_leaf3d	A2	3	leaf	2	116.87	-26.394
P_Ge_pu_A2_stem	A2	3	stem	2	125.59	-25.273
P_Ge_pu_A2_rhiz_1	A2	3	rhiz	2	-23.92	-26.656
P_Ge_pu_A2_rhiz_2	A2	3	rhiz	2	-25.69	-26.198
P_Ge_pu_A2_root_3	A2	3	root	2	-22.18	-26.575
P_Ge_pu_B2_leaf0d	B2	0	leaf	2	747.64	-26.394
P_Ge_pu_B2_leaf1d	B2	1	leaf	2	386.15	-26.394
P_Ge_pu_B2_leaf2d	B2	2	leaf	2	282.27	-26.394
P_Ge_pu_B2_leaf3d	B2	3	leaf	2	243.88	-26.394
P_Ge_pu_B2_stem	B2	3	stem	2	277.09	-25.273
P_Ge_pu_B2_ rhiz_1	B2	3	rhiz	2	-14.39	-26.656
P_Ge_pu_B2_ rhiz_2	B2	3	rhiz	2	-22.66	-26.198
P_Ge_pu_B2_root_4	B2	3	root	2	-24.96	-26.575
P_Ge_pu_C2_leaf0d	C2	0	leaf	2	443.59	-26.394
P_Ge_pu_C2_leaf1d	C2	1	leaf	2	95.69	-26.394
P_Ge_pu_C2_leaf2d	C2	2	leaf	2	99.07	-26.394
P_Ge_pu_C2_leaf3d	C2	3	leaf	2	88.23	-26.394
P_Ge_pu_C2_stem	C2	3	stem	2	125.07	-25.273
P_Ge_pu_C2_ rhiz_1	C2	3	rhiz	2	-18.32	-26.656
P_Ge_pu_C2_ rhiz_2	C2	3	rhiz	2	-22.82	-26.198
P_Ge_pu_C2_ root3d	C2	3	root	2	-22.79	-26.575

NAME	INDIV	DAY	TISSUE	PHENO-STAGE	δ13C	CONTROL δ13C
P_Pe_os_A1_leaf0d	A1	0	leaf	1	.	.
P_Pe_os_A1_leaf1d	A1	1	leaf	1	1171.1	-24.058
P_Pe_os_A1_leaf2d	A1	2	leaf	1	966.65	-24.058
P_Pe_os_A1_leaf3d	A1	3	leaf	1	699.48	-24.058
P_Pe_os_A1_stem	A1	3	stem	1	458.89	-25.085
P_Pe_os_A1_rhiz	A1	3	rhiz	1	38.89	-25.462
P_Pe_os_A1_root_1	A1	3	root	1	-17.82	-26.093
P_Pe_os_A1_fineroot	A1	3	fineroot	1	-26.23	-26.232
P_Pe_os_B1_leaf0d	B1	0	leaf	1	2006.29	-24.058
P_Pe_os_B1_leaf1d	B1	1	leaf	1	691.1	-24.058
P_Pe_os_B1_leaf2d	B1	2	leaf	1	458.17	-24.058
P_Pe_os_B1_leaf3d	B1	3	leaf	1	480.61	-24.058
P_Pe_os_B1_ stem	B1	3	stem	1	444.11	-25.085
P_Pe_os_B1_rhiz	B1	3	rhiz	1	-9.75	-25.462
P_Pe_os_B1_rhiz1_2	B1	3	rhiz	1	-16.29	-26.093
P_Pe_os_B1_ root	B1	3	root	1	-17.99	-26.232
P_Pe_os_C1_leaf0d	C1	0	leaf	1	769.9	-24.058
P_Pe_os_C1_leaf1d	C1	1	leaf	1	.	.
P_Pe_os_C1_leaf2d	C1	2	leaf	1	279.98	-24.058
P_Pe_os_C1_leaf3d	C1	3	leaf	1	249.08	-24.058
P_Pe_os_C1_ stem_1	C1	3	stem	1	151.72	-25.085
P_Pe_os_C1_rhiz_1	C1	3	rhiz	1	-19.31	-25.462
P_Pe_os_C1_rhiz1_3	C1	3	rhiz	1	-26.25	-26.251
P_Pe_os_C1_ root_1	C1	3	root	1	-22.66	-26.232
P_Pe_os_A2_leaf0d	A2	0	leaf	2	1326.3	-24.331
P_Pe_os_A2_leaf1d	A2	1	leaf	2	450.84	-24.331
P_Pe_os_A2_leaf2d	A2	2	leaf	2	347.45	-24.331
P_Pe_os_A2_leaf3d	A2	3	leaf	2	298.19	-24.331
P_Pe_os_A2_stem	A2	3	stem	2	361.38	-24.344
P_Pe_os_A2_rhiz_1	A2	3	rhiz	2	31.04	-26.318
P_Pe_os_A2_rhiz_2	A2	3	rhiz	2	6.46	-26.226
P_Pe_os_A2_root2+10	A2	3	root	2	-12.73	-26.288
P_Pe_os_B2_leaf0d	B2	0	leaf	2	1864.39	-24.331
P_Pe_os_B2_leaf1d	B2	1	leaf	2	961.97	-24.331
P_Pe_os_B2_leaf2d	B2	2	leaf	2	704.01	-24.331
P_Pe_os_B2_leaf3d	B2	3	leaf	2	684.11	-24.331
P_Pe_os_B2_ stem	B2	3	stem	2	391.77	-24.344
P_Pe_os_B2_rhiz1+2	B2	3	rhiz	2	14.21	-26.318
P_Pe_os_B2_rhiz3+4	B2	3	rhiz	2	-18.31	-26.226
P_Pe_os_B2_root1_7	B2	3	root	2	-9.08	-26.288
P_Pe_os_C2_leaf0d	C2	0	leaf	2	1527.06	-24.331
P_Pe_os_C2_leaf1d	C2	1	leaf	2	430.69	-24.331
P_Pe_os_C2_leaf2d	C2	2	leaf	2	324.19	-24.331
P_Pe_os_C2_leaf3d	C2	3	leaf	2	295.09	-24.331
P_Pe_os_C2_stem	C2	3	stem	2	594.19	-24.344
P_Pe_os_C2_rhiz_1	C2	3	rhiz	2	28	-26.318
P_Pe_os_C2_rhiz_2	C2	3	rhiz	2	-6.05	-26.226
P_Pe_os_C2_root_1	C2	3	root	2	25.37	-26.288

NAME	INDIV	DAY	TISSUE	PHENO-STAGE	δ13C	CONTROL δ13C
P_Ru_alpi_A1_leaf0d	A1	0	leaf	1	1328.89	-26.964
P_Ru_alpi_A1_leaf1d	A1	1	leaf	1	375.55	-26.964
P_Ru_alpi_A1_leaf2d	A1	2	leaf	1	192.1	-26.964
P_Ru_alpi_A1_leaf3d	A1	3	leaf	1	135.29	-26.964
P_Ru_alpi_A1_stem	A1	3	stem	1	91.65	-27.154
P_Ru_alpi_A1_rhiz_1	A1	3	rhiz	1	-6.9	-28.139
P_Ru_alpi_A1_rhiz_2	A1	3	rhiz	1	-22.74	-28.453
P_Ru_alpi_A1_root_2	A1	3	root	1	-14.74	-28.051
P_Ru_alpi_B1_leaf0d	B1	0	leaf	1	2352.84	-26.964
P_Ru_alpi_B1_leaf1d	B1	1	leaf	1	956.11	-26.964
P_Ru_alpi_B1_leaf2d	B1	2	leaf	1	529.92	-26.964
P_Ru_alpi_B1_leaf3d	B1	3	leaf	1	753.24	-26.964
P_Ru_alpi_B1_ stem	B1	3	stem	1	355.18	-27.154
P_Ru_alpi_B1_ rhiz_1	B1	3	rhiz	1	94.45	-28.139
P_Ru_alpi_B1_ rhiz_2	B1	3	rhiz	1	29.6	-28.453
P_Ru_alpi_B1_ root_1	B1	3	root	1	22.28	-28.051
P_Ru_alpi_C1_leaf0d	C1	0	leaf	1	713.82	-26.964
P_Ru_alpi_C1_leaf1d	C1	1	leaf	1	.	.
P_Ru_alpi_C1_leaf2d	C1	2	leaf	1	166.57	-26.961
P_Ru_alpi_C1_leaf3d	C1	3	leaf	1	169.6	-26.964
P_Ru_alpi_C1_ stem	C1	3	stem	1	98.15	-27.154
P_Ru_alpi_C1_ rhiz_1	C1	3	rhiz	1	0.55	-28.139
P_Ru_alpi_C1_ rhiz_2	C1	3	rhiz	1	-16.19	-28.453
P_Ru_alpi_C1_ root_2	C1	3	root	1	4.85	-28.051
P_Ru_alpi_A2_leaf0d	A2	0	leaf	2	2088.09	-25.829
P_Ru_alpi_A2_leaf1d	A2	1	leaf	2	332.15	-25.829
P_Ru_alpi_A2_leaf2d	A2	2	leaf	2	213.52	-25.829
P_Ru_alpi_A2_leaf3d	A2	3	leaf	2	222.42	-25.829
P_Ru_alpi_A2_stem	A2	3	stem	2	271.73	-25.769
P_Ru_alpi_A2_rhiz_1	A2	3	rhiz	2	72.32	-27.161
P_Ru_alpi_A2_rhiz_2	A2	3	rhiz	2	41.61	-27.601
P_Ru_alpi_A2_ root	A2	3	root	2	.	.
P_Ru_alpi_B2_leaf0d	B2	0	leaf	2	258.35	-25.829
P_Ru_alpi_B2_leaf1d	B2	1	leaf	2	9.28	-25.829
P_Ru_alpi_B2_leaf2d	B2	2	leaf	2	-2.51	-25.829
P_Ru_alpi_B2_leaf3d	B2	3	leaf	2	-10.97	-25.829
P_Ru_alpi_B2_ stem	B2	3	stem	2	110.7	-25.769
P_Ru_alpi_B2_ rhiz_1	B2	3	rhiz	2	21.99	-27.161
P_Ru_alpi_B2_ rhiz_2	B2	3	rhiz	2	1.86	-27.601
P_Ru_alpi_B2_ root	B2	3	root	2	.	.
P_Ru_alpi_C2_leaf0d	C2	0	leaf	2	1217.83	-25.829
P_Ru_alpi_C2_leaf1d	C2	1	leaf	2	387.47	-25.829
P_Ru_alpi_C2_leaf2d	C2	2	leaf	2	300.37	-25.829
P_Ru_alpi_C2_leaf3d	C2	3	leaf	2	214.35	-25.829
P_Ru_alpi_C2_ stem	C2	3	stem	2	387.81	-25.769
P_Ru_alpi_C2_ rhiz_1	C2	3	rhiz	2	197.15	-27.161
P_Ru_alpi_C2_ rhiz_2	C2	3	rhiz	2	166.53	-27.601
P_Ru_alpi_C2_ root	C2	3	root	2	.	.

ESM Fig. 1: CO_2 concentrations (ppm) in the plexiglass chamber during the ^{13}C pulse of three individuals each of the tall plant species, selected individuals were at stage 1 (first leaves visible) or at stage 2 (first leaves unfolded), respectively. Larger biomass at stage 2 (more photosynthetically active tissues) led to on average higher CO_2 plant uptake and faster CO_2 decline in the plexiglass chamber during pulsing.

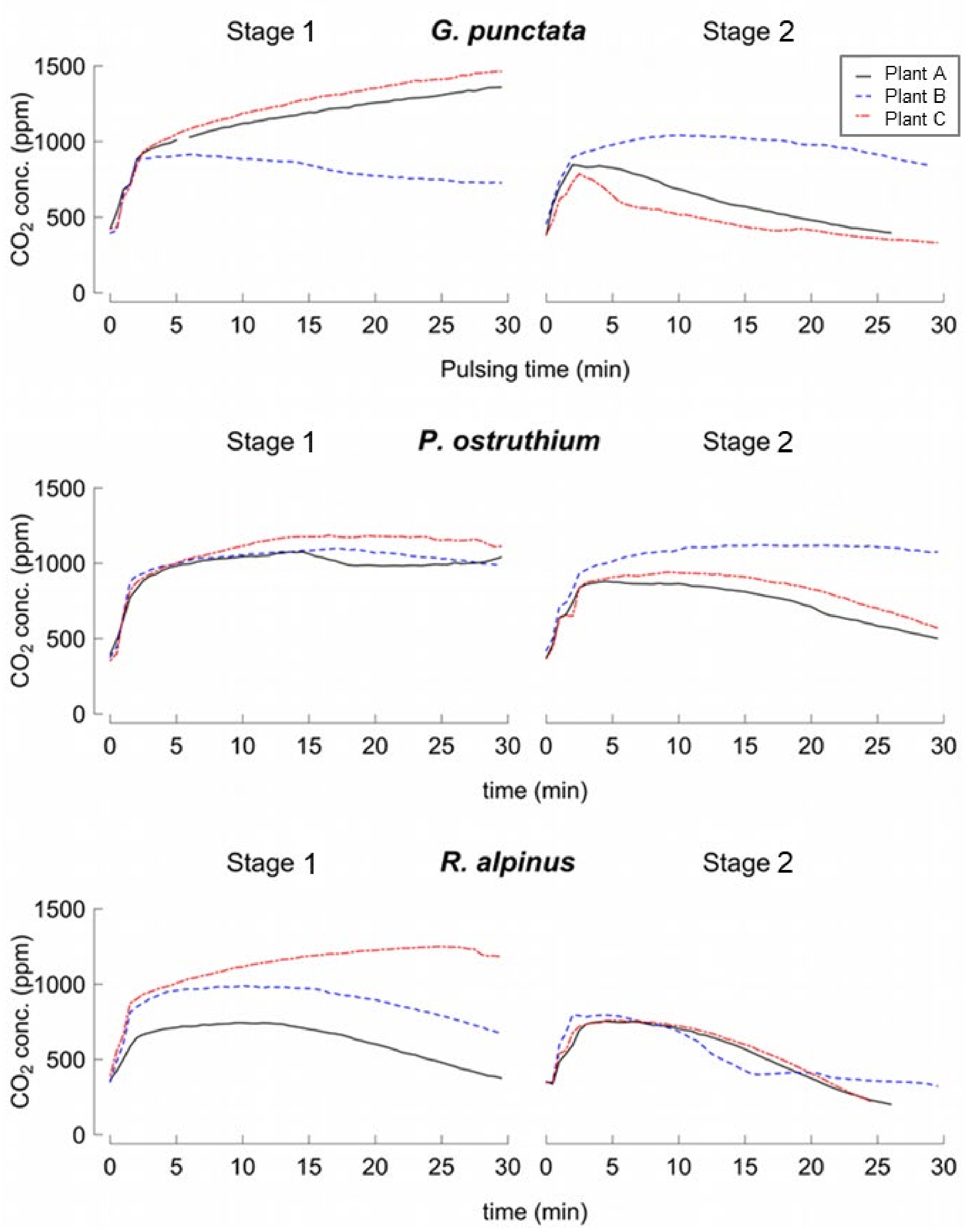

ESM Fig. 2: Stage 1 (left) and stage 2 (right) of an individual each of the tall herb *Gentiana punctata,* selected for the ^{13}C pulsing. White rim shows the position of the plexiglass chamber

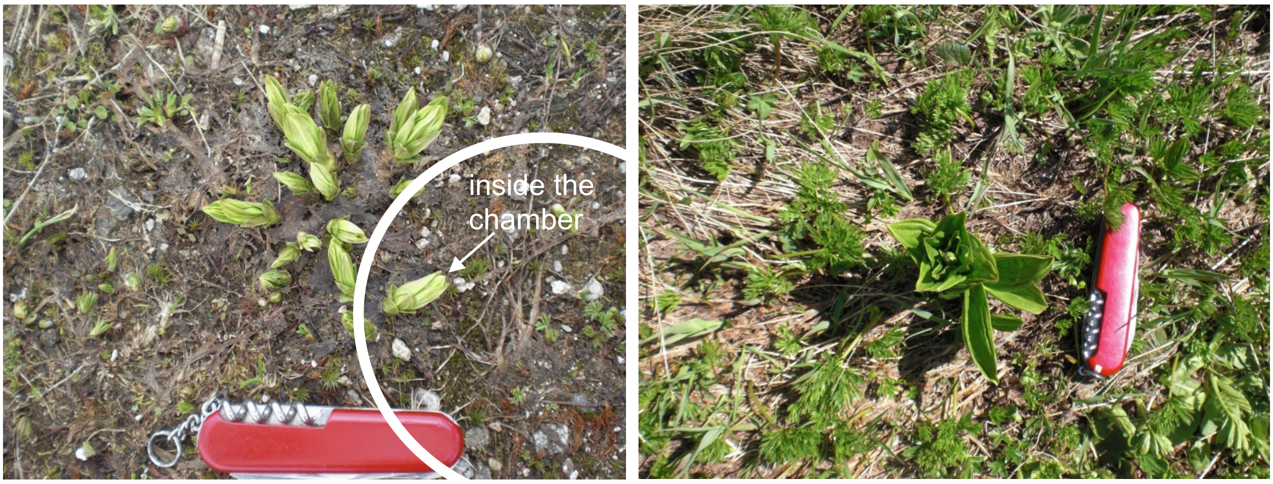

Supplement: Supplementary file 1 — Supplementary file1 (PDF 779 kb) [file 442_2021_4950_MOESM1_ESM.pdf]
